# Supplementary material for: Knowledge transfer-driven estimation of knee moments and ground reaction forces from smartphone videos via temporal-spatial modeling of augmented joint kinematics
Source: PLoS One. 2025 Nov 7;20(11):e0335257. doi: 10.1371/journal.pone.0335257 (PMC12594377; doi:10.1371/journal.pone.0335257)
Supplement: S1 File — File containing additional equations and tables. (PDF) [file pone.0335257.s001.pdf]

Supplementary Materials

Multi Fusion Module (MFM): We combine multiple fusion modules through late weighted fusion (LWF) to create MFM (Fig S1). Specifically, our MFM consists of multi-head attention fusion (MHAF), weighted feature fusion (WFF), tensor multiplication fusion (TMF), and late weighted fusion (LWF).

(i) Multi-Head Attention Fusion (MHAF): Multi-head self-attention module can help to model the relationship between the features of a sequence. We apply multi-head self-attention to the multi-modal concatenated features, which may provide us with important relationships between different modalities. First, concatenated features $X_{student}^{concat,bi-lstm,gcn}$ (Equation 25 of the paper) are gone through linear projection to create Query (Q), Key (K), and Value (V) matrices. If the linear projections for transforming input sequences are W_Q, W_K, W_V, then Q, K, and V can be realized using Equations S1, S2, and S3.

$$Q = X_{student}^{concat,bi-lstm,gcn} W_Q \tag{S1}$$
$$K = X_{student}^{concat,bi-lstm,gcn} W_K \tag{S2}$$
$$V = X_{student}^{concat,bi-lstm,gcn} W_V \tag{S3}$$

Here, $X_{student}^{concat,bi-lstm,gcn} \in \mathbb{R}^{B \times \Delta T \times 3C}$, $W_Q, W_K, W_V \in \mathbb{R}^{3C \times d}$, and $Q, K, V \in \mathbb{R}^{B \times \Delta T \times d}$, $3C$ is the total number of concatenated features, d is the dimension of linear projection. The attention-weighted matrix can be found using Equation S4.

$$Attention(Q, K, V) = Softmax(QK^T/\sqrt{d})V \tag{S4}$$

Multiple attention heads are applied to understand different types of relationships among the input sequences. Output from multiple attention heads is then concatenated together and projected with a linear layer to obtain the original space.

$$X_{student}^{concat,mhaf} = Concat(Attention(Q_i, K_i, V_i)_{i=1}^{h})W_o \tag{S5}$$

Here, h is the number of heads, $W_o \in \mathbb{R}^{d.h}$, $d = 3C/h$.

(ii) Weighted Feature Fusion (WFF): The inclusion of features from multiple modalities does not necessarily guarantee optimal performance. In order to prioritize the most relevant features, we propose the utilization of a weighted feature fusion technique for the integration of multi-modal data. If the concatenated student features are $X_{student}^{concat,bi-lstm,gcn}$, then the output from the WFF module can be derived utilizing Equation S6 and S7.

$$W_{feature} = Sigmoid(FC(X_{student}^{concat,bi-lstm,gcn})) \tag{S6}$$
$$X_{student}^{concat,wff} = W_{feature} * X_{student}^{concat,bi-lstm,gcn} \tag{S7}$$

(iii) Tensor Multiplication Fusion (TMF): In order to incorporate additional context into multi-modal features, we employ element-wise tensor multiplication. The capture of interaction and relationships between several modalities can be valuable. The given approach effectively emphasizes the prevalent characteristics observed in several modalities while suppressing those that are deemed less important. TMF operation can be realized using Equation S8.

$$X_{student}^{concat,tmf} = X_{student,jcv}^{concat,bi-lstm,gcn} * X_{student,jca}^{concat,bi-lstm,gcn} * X_{student,jcp}^{concat,bi-lstm,gcn} \tag{S8}$$

Here $X_{student,jcv}^{concat,bi-lstm,gcn}$, $X_{student,jca}^{concat,bi-lstm,gcn}$, $X_{student,jcp}^{concat,bi-lstm,gcn}$ are derived by indexing the previously concatenated multi-modal features of $X_{student}^{concat,bi-lstm,gcn}$.

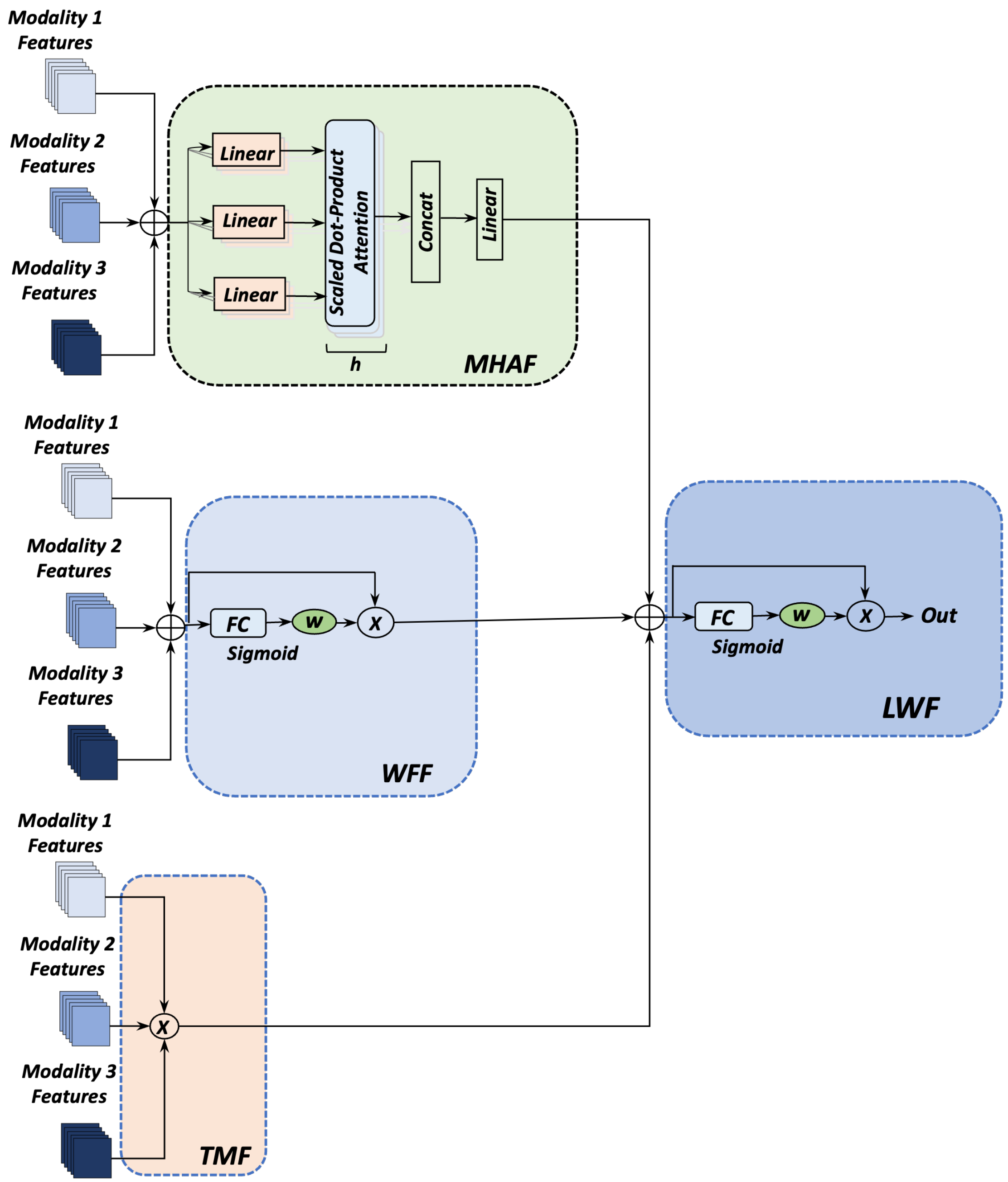

Fig S1. Detailed architecture of our MFM module

(iv) Late Weighted Fusion (LWF): Since we integrate various fusion strategies in our model, it is necessary to further enhance the significance of the features from each fusion module. To accomplish this objective, we propose a late-weighted fusion module. Specifically, the LWF can be implemented utilizing Equations S9, S10, and S11.

$$X_{student}^{concat,lwf} = [X_{student}^{concat,mhaf}, X_{student}^{concat,wff}, X_{student}^{concat,tmf}] \tag{S9}$$

$$W_{feature} = Sigmoid(FC(X_{student}^{concat,lwf})) \tag{S10}$$

$$X_{student}^{concat,mfm} = W_{feature} * X_{student}^{concat,lwf} \tag{S11}$$

Table S1. Comparison of Different Fusion Modules in the Student Model. A detailed evaluation of how different fusion techniques impact the estimation performance when applied to the student model.

**Fusion Module**	**NRMSE(%)**	**PCC**
TMF	5.28 ± 0.56**	0.939 ± 0.015**
WFF	4.91 ± 0.53	0.945 ± 0.014
MHAF	5.05 ± 0.53	0.944 ± 0.013
WFF+MHAF	4.91 ± 0.52	0.945 ± 0.014
LWF+WFF+MHAF	4.93 ± 0.59	0.946 ± 0.015
WFF+TMF	4.97 ± 0.52	0.943 ± 0.015
LWF+WFF+TMF	4.99 ± 0.54	0.943 ± 0.014
MHAF+TMF	5.05 ± 0.51	0.943 ± 0.013
LWF+MHAF+TMF	5.02 ± 0.58	0.943 ± 0.015
WFF+MHAF+TMF	4.98 ± 0.52	0.945 ± 0.014
**LWF+WFF+MHAF+TMF**	**4.85 ± 0.62**	**0.946 ± 0.015**

The bold numbers indicate the highest performance in NRMSE and PCC. ** and * indicate a significant difference in NRMSE and PCC between LWF+WFF+MHAF+TMF and the other combination of fusion modules, for $p < 0.01$ and $p < 0.05$, respectively.

Table S2. Mean and standard deviation of NRMSE and PCC for all kinetics components using KD (Vanilla) and KD (Vanilla and layer loss) with different α values.

α	KD (Vanilla)		KD (Vanilla+Layer Loss)	
	NRMSE(%)	PCC	NRMSE(%)	PCC
0.10	4.91 ± 0.57*	0.945 ± 0.014**	4.93 ± 0.56**	0.945 ± 0.013**
0.20	4.86 ± 0.56	0.946 ± 0.015**	4.94 ± 0.51	0.945 ± 0.015
0.30	4.91 ± 0.56*	0.946 ± 0.014**	4.85 ± 0.53	0.946 ± 0.014**
0.40	4.91 ± 0.54*	0.945 ± 0.016**	4.89 ± 0.56*	0.946 ± 0.014**
0.50	4.89 ± 0.59**	0.946 ± 0.014**	4.87 ± 0.53*	0.946 ± 0.013**
0.60	4.85 ± 0.50	0.946 ± 0.014**	4.89 ± 0.56*	0.945 ± 0.015**
0.70	4.92 ± 0.51*	0.945 ± 0.014**	4.89 ± 0.57	0.945 ± 0.014**
0.80	4.88 ± 0.52*	0.946 ± 0.014**	4.93 ± 0.53**	0.946 ± 0.015**
0.90	4.91 ± 0.48*	0.946 ± 0.013**	4.89 ± 0.52**	0.945 ± 0.013**

** and * indicate a significant difference in NRMSE and PCC between Joint Dynamics+MFM+Gate+BiLSTM+Attn-GCN model with the proposed knowledge transfer and Joint Dynamics+MFM+Gate+BiLSTM+Attn-GCN model with vanilla and vanilla+layer loss KD with different weighting components, for $p < 0.01$ and $p < 0.05$, respectively.

Table S3. Comparison of Different Fusion Modules in the Teacher Model. A detailed analysis of the performance of different fusion strategies when applied to the teacher model.

**Fusion Module**	**NRMSE(%)**	**PCC**
TMF	3.86 ± 0.43**	0.968 ± 0.009**
WFF	3.87 ± 0.49**	0.969 ± 0.009**
MHAF	3.84 ± 0.47*	0.969 ± 0.010
WFF+MHAF	3.78 ± 0.45*	0.970 ± 0.010
LWF+WFF+MHAF	3.66 ± 0.48	0.972 ± 0.009
WFF+TMF	3.91 ± 0.49**	0.969 ± 0.009**
LWF+WFF+TMF	3.86 ± 0.45**	0.968 ± 0.009**
MHAF+TMF	3.79 ± 0.45**	0.970 ± 0.009
LWF+MHAF+TMF	3.71 ± 0.49	0.971 ± 0.008
WFF+MHAF+TMF	3.79 ± 0.45	0.969 ± 0.009
**LWF+WFF+MHAF+TMF**	3.63 ± 0.47	0.973 ± 0.009

The bold numbers indicate the highest performance in NRMSE and PCC. ** and * indicate a significant difference in NRMSE and PCC between LWF+WFF+MHAF+TMF and the other combination of fusion modules, for $p < 0.01$ and $p < 0.05$, respectively.
