# Supplementary material for: Knowledge transfer-driven estimation of knee moments and ground reaction forces from smartphone videos via temporal-spatial modeling of augmented joint kinematics
Source: PLoS One. 2025 Nov 7;20(11):e0335257. doi: 10.1371/journal.pone.0335257 (PMC12594377; doi:10.1371/journal.pone.0335257)
Supplement: Attachment — Submitted filename: Rebuttal Letter.pdf [file pone.0335257.s002.pdf]

Editor Comments: Please be sure to address all reviewer comments for clarifications and additional details. If at all possible, including at least a small pilot study to diversify the subject pool would strengthen the paper.

Response: We thank the editor for the thoughtful suggestion to include a pilot study with a more diverse subject pool and for emphasizing on addressing all reviewer comments. We have responded to all reviewer comments with extensive clarifications and details.

The present work is based on a publicly available dataset that only includes young, healthy male participants. Since we did not control the data collection process, it is not possible to expand this dataset with additional pilot experiments. Moreover, conducting a new pilot study would require IRB approval and subject recruitment, both of which involve substantial administrative and logistical processes that cannot be readily implemented within the scope of the present study. For these reasons, adding a pilot study is not feasible here, though we fully agree and emphasize in the manuscript that future investigations should recruit more diverse populations to enhance generalizability.

Reviewers' comments:

Reviewer's Responses to Questions

Comments to the Author

1. Is the manuscript technically sound, and do the data support the conclusions?

The manuscript must describe a technically sound piece of scientific research with data that supports the conclusions. Experiments must have been conducted rigorously, with appropriate controls, replication, and sample sizes. The conclusions must be drawn appropriately based on the data presented.

Reviewer #1: Yes

Reviewer #2: Yes

Response: We thank both reviewers for recognizing that the manuscript is technically sound and that the data appropriately support the conclusions.

---

2. Has the statistical analysis been performed appropriately and rigorously?

Reviewer #1: No

Reviewer #2: Yes

Response:

We thank both reviewers for their feedback regarding the statistical analysis. We carefully verified our procedures and confirm that all analyses were performed appropriately and rigorously. Specifically, we conducted a Repeated-Measures Analysis of Variance (ANOVA), followed by pairwise post hoc comparisons with Bonferroni correction for both NRMSE and PCC values, using IBM SPSS Statistics (Version 29, IBM, Armonk, NY). In the original manuscript, we mistakenly wrote "least significant difference (LSD)" instead of "Bonferroni correction," which may have caused confusion. This has been corrected in the revised manuscript. We used $p < 0.05$ as the primary threshold for statistical significance, and additionally reported $p < 0.01$ to highlight cases with stronger statistical evidence.

---

3. Have the authors made all data underlying the findings in their manuscript fully available?

The PLOS Data policy requires authors to make all data underlying the findings described in their manuscript fully available without restriction, with rare exception (please refer to the Data Availability Statement in the manuscript PDF file). The data should be provided as part of the manuscript or its supporting information, or deposited to a public repository. For example, in addition to summary statistics, the data points behind means, medians and variance measures should be available. If there are restrictions on publicly sharing data—e.g. participant privacy or use of data from a third party—those must be specified.

Reviewer #1: Yes

Reviewer #2: Yes

Response: We thank both reviewers for confirming that all data underlying our findings have been made fully available in accordance with PLOS ONE's data policy.

---

4. Is the manuscript presented in an intelligible fashion and written in standard English?

PLOS ONE does not copyedit accepted manuscripts, so the language in submitted articles must be clear, correct, and unambiguous. Any typographical or grammatical errors should be corrected at revision, so please note any specific errors here.

Reviewer #1: Yes

Reviewer #2: Yes

<u>Response:</u> We thank both reviewers for acknowledging that the manuscript is clearly written, intelligible, and presented in standard English.

---

5. Review Comments to the Author

Reviewer #1: General Overview
Overall, this study presents a smartphone-based kinetics estimation pipeline that integrates augmented kinematic data, knowledge transfer, and spatio-temporal modeling. The diverse machine learning techniques alongside biomechanical insights has led to improved estimation performance, which the authors have validated through rigorous ablations.

However, I have a major concern regarding the generalizability of the proposed pipeline. Although the study includes data from seventeen subjects, the variation in gender, age, height, and weight is extremely limited. This raises doubts about the robustness of the estimation performance, particularly when tested on subjects with gait characteristics similar to those in the training set.
Due to the narrow subject diversity, applying this technology to patients with irregular or pathological gait patterns may result in poor estimation accuracy and unreliable clinical interpretation.

<u>Response:</u> We fully agree with the mentioned limitation of the study. We have now further revised with additional context to highlight the limitation of the study for the readers. Specifically, we added the following paragraph at the ‘Limitations’ subsection of the “Results and Discussion” section.

“(1) the dataset used in this study consisted of a relatively homogeneous group of young, healthy males with limited variation in age, height, and weight. This lack of demographic and anthropometric diversity may restrict the generalizability of the deep learning model to broader populations, such as females, older adults, or patients with gait impairments. While utilizing leave-one-subject-out cross-validation mitigates some of the generalization issue for unseen subjects, still a large cohort of subject training is needed for clinical deployment”

Additionally, the term “joint dynamics” does not seem appropriate for describing the input to the proposed framework. Dynamics typically refers to properties involving mass or inertia, which are not directly captured by IMUs. I recommend using alternative terms, such as “joint kinematics,” to avoid confusion. (Page 3, Line 60; Page 4, Line 85)

Response: We agree with the reviewer that the term “joint dynamics” could cause confusion, as dynamics typically refers to mass or inertia-related properties. Therefore, we have replaced “joint dynamics” with “augmented joint kinematics.” We use the qualifier “augmented” to clarify that our features are based on joint center positions, velocities, and accelerations, rather than joint angles as conventionally defined in biomechanics. This terminology has been updated consistently throughout the manuscript, including in the name of the models.

Introduction

While IMUs can be used to predict inertial information such as joint moments, they do not directly provide this information. I recommend using more precise terminology—such as “predict” instead of “provide”—to avoid the confusion.

Response: *We sincerely thank the reviewer for pointing this out. We agree that the original sentence seems to be confusing. To address this, we have revised the sentence to improve clarity.* Specifically, we removed the previous sentence and added the following sentence in the manuscript: “In contrast, IMUs can measure the segment-level acceleration and angular velocities, which can then be used to predict joint moments.”

Method

I recommend that the authors include quantitative results comparing their smartphone-based motion tracking to ground-truth kinematic data. Providing motion tracking metrics (e.g., RMSE of joint angle predictions) would help readers understand the system’s inherent accuracy. This

distinction is crucial for understanding whether the observed kinetics estimation errors come from the model itself or limitations in the motion tracking input.

Response: We thank the reviewer for this valuable suggestion. The dataset used in this study provides optical motion capture marker trajectories and 2D joint keypoints from two smartphone cameras; however, it does not include musculoskeletal modeling outputs such as joint angles from inverse kinematics. Since no ground-truth joint kinematics were provided in the dataset, we cannot directly compute RMSE of joint angles between smartphone-based tracking and marker-based reference data. Nevertheless, prior studies have reported that OpenPose-based tracking introduces measurable errors relative to gold-standard optical motion capture, and other works have shown that even kinetics predicted from MoCap-derived kinematics exhibit non-negligible estimation errors. These findings suggest that the kinetics estimation error observed in our framework reflects a combination of (i) uncertainty in smartphone-based input tracking and (ii) inherent model-related limitations. We have clarified this point in the "Results and Discussion" section of the revised manuscript.

The manuscript states that 2D joint positions were normalized to the subject's height to mitigate size variability. However, joint kinetics inherently depend on body segment lengths and mass distribution. How did the authors recover the true scale required for joint moment estimation?

Response: We thank the reviewer for pointing this out and agree that further clarification is helpful. In our study, KAM and KFM are provided in dimensionless form, normalized by body weight and body height, and expressed as percentages in the original dataset. We normalized GRFs by subject body weight. These normalization strategies ensure that inter-subject variability due to body size or mass is reduced, enabling the deep learning model to learn subject-independent mappings between 2-D joint center input and kinetics output. Importantly, because the ground truth data are already normalized, the model does not require explicit knowledge of the subject's absolute body weight or height at inference. If subject-specific values in physical units are needed, they can be recovered post-prediction by rescaling the normalized outputs with the individual's body weight and height. This approach balances generalizability across subjects while still allowing biomechanically meaningful scaling when subject-specific information is available. We have provided these explanations in the *Dataset*

Description section of the revised manuscript to help readers better understand this point.

It is unclear how the authors combined joint keypoints extracted from two different smartphone videos. Was triangulation or another 3D reconstruction method used? This step should be described in the methodology section for reproducibility.

<u>Response:</u> We thank the reviewer for the feedback. We utilized 2D joint center positions from both cameras and concatenated them into a single feature vector; hence, triangulation or 3D reconstruction was not applied in this work.

The authors mention that accelerations and angular velocities were derived from positional data. Since IMUs directly measure these quantities, it would be insightful to compare the derived signals with true IMU measurements. I suggest including a comparison plot of smartphone-derived versus IMU-recorded accelerations and angular velocities. This would help readers evaluate how much signal error propagates through the pipeline.

<u>Response:</u>

We thank the reviewer for this suggestion. Our input consists of 2D joint center positions, from which we derive linear velocities and accelerations at the joint centers by finite differencing. These are not equivalent to IMU measurements, since IMUs mounted on body segments capture segment-specific linear acceleration (including gravity) and angular velocity in 3D space, which cannot be reconstructed from 2D joint center trajectories without knowledge of segment orientation and sensor pose. For this reason, a direct comparison between derived joint-center dynamics and IMU recordings is not feasible in our dataset.

Deriving acceleration and angular velocity from position data is prone to noise. Did the authors apply any signal processing techniques (e.g., low-pass filtering, smoothing, or delaying) to address this? A brief explanation of the data preprocessing pipeline would improve transparency.

<u>Response:</u> We thank the reviewer for raising this important concern. In our current implementation, we derived velocities and accelerations from joint-center positions using finite differencing without applying additional low-pass filtering. We provide the pre-processing details in the 'proposed approach' section (a) Joint center position, velocity, and acceleration).

We acknowledge that this may introduce high-frequency noise; however, recurrent sequence models such as GRUs and LSTMs leverage temporal context, making their predictions more robust to small, noisy variations in the data [1,2].

At the same time, we agree that excessive noise could affect performance, and we have added this point as a limitation in the revised manuscript, noting that future work will investigate noise reduction techniques to further improve robustness.

Specifically, we include the following sentences in our Limitation and Future Work section.

"(3) joint center velocities and accelerations were generated using the finite differencing method without applying any low-pass filtering or smoothing, which may introduce high-frequency noise into the input. Although recurrent models such as LSTMs or GRUs can leverage temporal context and are relatively robust to small amounts of noise [1,2], excessive noise can still degrade model performance."

"To mitigate the effect of high-frequency noise introduced by the finite differencing method, future work could incorporate signal processing techniques such as low-pass filtering or smoothing or explore noise-robust training strategies."

1. Yeo K. Short note on the behavior of recurrent neural network for noisy dynamical system. arXiv preprint arXiv:190405158. 2019.
2. Rubinstein B. It's a super deal–train recurrent network on noisy data and get smooth prediction free. arXiv preprint arXiv:220604215. 2022.

Does the proposed pipeline require the subject's body weight for joint kinetics estimation? If so, how is this information incorporated into the model? If not, can authors comment on how the absence of body weight information might impact the accuracy of the estimated forces and moments.

Response: We thank the reviewer for raising this concern. Our pipeline does not require explicit body weight during inference, since the ground truth kinetics in the dataset are already provided in normalized form (scaled by body weight and/or height). As a result, the absence of body weight information at inference is not expected to affect model accuracy in the normalized domain. When subject-specific values in physical units are

needed, the normalized predictions can be rescaled using the individual's body weight and height.

We already clarified this in 'Data Description' section to help the readers.

Regarding comparisons with state-of-the-art (SOTA) models: did the authors evaluate all models using the exact same dataset and evaluation protocol? Clarifying this in the manuscript will enhance the fairness of the comparative results.

Response: We thank the reviewer for raising this important point. We have clarified in the revised manuscript that all models, including the baselines and our proposed method, were evaluated on the same dataset, preprocessing steps, and leave-one-subject-out cross-validation protocol to ensure fairness of comparison. We also note that full implementations of all baseline models and our proposed framework have been made publicly available in our GitHub repository for transparency and reproducibility.

Figure 2: For improved readability, I suggest labeling components like attention score, attention-weighted features, and final outputs directly along the arrows with their corresponding symbols in the diagram.

Response: We thank the reviewer for this helpful suggestion. We slightly modify Fig. 2 to label attention score, attention weighted features, and final output. Additionally, we also include a figure (Fig S1) in the supplementary materials to better explain the MFM module to the readers.

Figure 3: Please include the units for moment and force values.

Response: We thank the reviewer for pointing this out. We have updated Figure 3 to include the units: KAM and KFM in %BW·BH, and GRFs in BW. We also include the following paragraph in the pre-processing pipeline to clarify how we derive the unit of joint moments and GRFs.

"KAM and KFM are provided in dimensionless form, normalized by body weight and body height, and expressed as percentages in the original dataset. We normalized GRFs by subject body weight."

Discussion

The authors mentioned that the smartphone-based tracking has inherent limitations due to camera placement. From the utility perspective when used

in real clinical settings, how many gait cycles can the proposed framework capture within the reported error boundary (NRMSE of 4.68)?

Response: We thank the reviewer for this important question. To clarify, we have added the following paragraph to the *Results and Discussion* section:

"Our dataset was collected on a treadmill, where continuous gait cycles were captured under controlled conditions. The reported NRMSE of 4.68 reflects performance across all gait cycles included in our analysis, not just a single cycle, suggesting that comparable accuracy can be expected across repeated cycles as long as they are reliably recorded by smartphones. In real-world clinical environments, overground walking is more common, where factors such as walkway length, camera placement, and patient mobility may influence how many complete gait cycles can be recorded. Provided that full cycles are accurately processed by OpenPose, we anticipate similar performance within the reported error boundary. Future validation under overground and clinical conditions will be important to confirm robustness across multiple gait cycles and practical deployment scenarios."

Also, would it be enough to get NRMSE of 4.68 for most of the clinical applications? I would be more helpful to include such information in the discussion part with some references.

Response: We thank the reviewer for pointing this out. We included the following paragraph in our "Results and Discussion" section to provide more details on this.

"While a performance gap remains between the teacher (NRMSE 3.63) and the proposed model (NRMSE 4.68), this reflects a trade-off between prediction accuracy and practical feasibility, as the proposed model requires only smartphone video input without additional IMU sensors. Despite outperforming existing methods, it is challenging to directly interpret the clinical significance of a specific NRMSE value, as no formal thresholds have been established for joint kinetics to the best of our knowledge. The acceptability of an error margin depends strongly on the clinical context, such as whether the method is applied for early detection, longitudinal monitoring, or individualized clinical decision-making. Nevertheless, our model achieved an NRMSE of 4.68 alongside a correlation coefficient of 0.957 with ground-truth kinetics, which indicates very strong agreement according to established guidelines for correlation interpretation [1,2]. These results suggest that the proposed framework provides highly reliable

estimates of joint kinetics, though prospective validation studies will be required to establish task-specific thresholds for clinical acceptability.”

1. Mukaka MM. A guide to appropriate use of correlation coefficient in medical research. Malawi medical journal. 2012;24(3):69–71.

2. Schober P, Boer C, Schwarte LA. Correlation coefficients: appropriate use and interpretation. Anesthesia & analgesia. 2018;126(5):1763–1768.

Reviewer #2: This is a well-written and technically robust manuscript that presents an innovative method to estimate knee joint moments (KAM, KFM) and 3D ground reaction forces (GRFs) using only smartphone video data. The authors introduce a multi-modal knowledge transfer approach from a teacher model (with IMU and video data) to a student model (video-only) for improving estimation accuracy. The proposed method significantly enhances accessibility and usability for gait analysis and rehabilitation purposes, potentially translating to real-world clinical settings.

1. The dataset includes only young healthy male subjects. The lack of diverse populations (e.g., females, older adults, patients) limits generalizability.

Response: We agree with the reviewer regarding the limitation. We add a comprehensive paragraph regarding this on the ‘Future Work’ subsection.

2. As acknowledged, 2D joint estimation quality from OpenPose can degrade in real-world conditions (e.g., occlusions, variable lighting), which can impact model performance.

Response: We agree with the reviewer, and this limitation is acknowledged and retained in the revised manuscript.

3. While the model outperforms others, the architecture may still be computationally intensive for real-time use on mobile devices without further optimization.

Response: We agree with the reviewer’s feedback and have explicitly added this point as a limitation in the revised manuscript with the following sentence: “(4) although the proposed architecture outperforms state-of-the-

art models in accuracy and efficiency, it may still be too computationally intensive for real-time use on mobile devices without further optimization."

4. Although the knowledge transfer reduces the performance gap, it's still notable (NRMSE of 4.68 vs. 3.63), and more discussion on real-world acceptability of this gap would be helpful.

<u>Response:</u> We thank reviewer for insightful suggestions. We add the following paragraph in "Results and Discussion" section to take care of this.

"While a performance gap remains between the teacher (NRMSE 3.63) and the proposed model (NRMSE 4.68), this reflects a trade-off between prediction accuracy and practical feasibility, as the proposed model requires only smartphone video input without additional IMU sensors. Despite outperforming existing methods, it is challenging to directly interpret the clinical significance of a specific NRMSE value, as no formal thresholds have been established for joint kinetics to the best of our knowledge. The acceptability of an error margin depends strongly on the clinical context, such as whether the method is applied for early detection, longitudinal monitoring, or individualized clinical decision-making. Nevertheless, our model achieved an NRMSE of 4.68 alongside a correlation coefficient of 0.957 with ground-truth kinetics, which indicates very strong agreement according to established guidelines for correlation interpretation [1,2]. These results suggest that the proposed framework provides highly reliable estimates of joint kinetics, though prospective validation studies will be required to establish task-specific thresholds for clinical acceptability."

1. Mukaka MM. A guide to appropriate use of correlation coefficient in medical research. Malawi medical journal. 2012;24(3):69–71.

2. Schober P, Boer C, Schwarte LA. Correlation coefficients: appropriate use and interpretation. Anesthesia & analgesia. 2018;126(5):1763–1768.

5. While the translational potential is mentioned, real-world deployment strategies, clinical thresholds for acceptability, or use in longitudinal tracking are not deeply discussed.

<u>Response:</u> We thank the reviewer for the valuable suggestions. We integrated those discussions in our 'Results and Discussion' section. More specifically, we added the following paragraph for each point:

real-world deployment strategies:

“To facilitate real-time deployment in clinical and home settings with mobile devices, the architecture can be compressed with advanced deep learning methods such as pruning, quantization, or reducing the number of units in the selected layers. Such strategies are essential to ensure that the framework can be realistically adopted in clinics, where computing resources and time are limited.”

clinical thresholds for acceptability:

“While a performance gap remains between the teacher (NRMSE 3.63) and the proposed model (NRMSE 4.68), this reflects a trade-off between prediction accuracy and practical feasibility, as the proposed model requires only smartphone video input without additional IMU sensors. Despite outperforming existing methods, it is challenging to directly interpret the clinical significance of a specific NRMSE value, as no formal thresholds have been established for joint kinetics to the best of our knowledge. The acceptability of an error margin depends strongly on the clinical context, such as whether the method is applied for early detection, longitudinal monitoring, or individualized clinical decision-making. Nevertheless, our model achieved an NRMSE of 4.68 alongside a correlation coefficient of 0.957 with ground-truth kinetics, which indicates very strong agreement according to established guidelines for correlation interpretation [1,2]. These results suggest that the proposed framework provides highly reliable estimates of joint kinetics, though prospective validation studies will be required to establish task-specific thresholds for clinical acceptability.”

1. Mukaka MM. A guide to appropriate use of correlation coefficient in medical research. Malawi medical journal. 2012;24(3):69–71.

2. Schober P, Boer C, Schwarte LA. Correlation coefficients: appropriate use and interpretation. Anesthesia & analgesia. 2018;126(5):1763–1768.

use in longitudinal tracking:

“Moreover, longitudinal studies are needed to assess the ability of the framework to track changes in KAM, KFM, and GRFs over time. This would allow clinicians to monitor patient progress and treatment response over weeks or months, making the framework more useful in rehabilitation and clinical contexts.”

6. Future studies should test on older adults, females, and patients with gait abnormalities to assess model generalizability.

7. Perform experiments under various lighting, occlusion, and video quality conditions to evaluate robustness.

8. Explore lightweight versions of the model for edge computing or real-time deployment on smartphones.

9. Consider testing how well the model tracks changes in KAM/KFM/GRFs over time, particularly in clinical recovery settings.

10. Provide insights into which joint features (e.g., hip vs. ankle) most influence kinetic estimation to improve interpretability for clinicians.

Response: We thank reviewers to provide these valuable suggestions. We incorporated these suggestions into the revised manuscript. Specifically, we added the following paragraph to the '*Future Work'* of "Results and Discussion" Section:

"Future studies should address these limitations by incorporating a diverse population comprising different age groups, sexes, and clinical populations to enhance generalizability. To mitigate the effect of high-frequency noise introduced by the finite differencing method, future work could incorporate signal processing techniques such as low-pass filtering or smoothing or explore noise-robust training strategies. Another key step is to evaluate model robustness under more realistic smartphone recording conditions, including occlusion, varying lighting, and camera quality. To facilitate real-time deployment in clinical and home settings with mobile devices, the architecture can be compressed with advanced deep learning methods such as pruning, quantization, or reducing the number of units in the selected layers. Such strategies are essential to ensure that the framework can be realistically adopted in clinics, where computing resources and time are limited. Moreover, longitudinal studies are needed to assess the ability of the framework to track changes in KAM, KFM, and GRFs over time. This would allow clinicians to monitor patient progress and treatment response over weeks or months, making the framework more useful in rehabilitation and clinical contexts. Another important direction is to investigate which joint centers contribute most to estimating knee moments. For example, KAM is

likely influenced most by the relative positions of the hip, knee, and ankle in the frontal plane, whereas KFM may depend more on the positions of the knee and ankle in the sagittal plane. Studying which joint centers carry the most weight in the prediction would make the model easier for clinicians to interpret and apply."

11. Grammar: The writing is clear and grammatically sound.

Response: We thank reviewers for the positive feedback.

12. Figures: Ensure all figures (especially Figs 1–3) are high-resolution and clearly labeled. They are essential for understanding model structure and performance.

Response: We thanked reviewer for the suggestion. We made all the figures high-resolution and clearly labelled.

13. Acronyms: Ensure all acronyms are spelled out at first use (e.g., GRF, GCN, Bi-LSTM).

Response: We thank the reviewer for pointing this out. We have carefully revised the manuscript to ensure that all acronyms (e.g., GRF, GCN, Bi-LSTM, PCC, NRMSE) are defined at first mention.

14. Ethical Statement: Appropriately addressed; no issues.

Response: We thank the reviewer for confirming that the ethical statement is clear and appropriate.
